# Markers for predicting the efficacy of beta-blockers in vasovagal syncope management in children: A mini-review

**DOI:** 10.3389/fcvm.2023.1131967

**Published:** 2023-03-08

**Authors:** Jing Wang, Xueqin Liu, Hongfang Jin, Junbao Du

**Affiliations:** ^1^ Department of Pediatrics, Peking University First Hospital, Beijing, China; ^2^ Key Laboratory of Molecular Cardiovascular Sciences, Ministry of Education, Beijing, China

**Keywords:** vasovagal syncope, beta-adrenergic receptor blockers, individualized management, children, prediction

## Abstract

Vasovagal syncope (VVS) is a common subtype of neurally mediated syncope. It is prevalent in children and adolescents, and critically affects the quality of life of patients. In recent years, the management of pediatric patients with VVS has received extensive attention, and β-blocker serves as an important choice of the drug therapy for children with VVS. However, the empirical use of β-blocker treatment has limited therapeutic efficacy in patients with VVS. Therefore, predicting the efficacy of β-blocker therapy based on biomarkers related to the pathophysiological mechanism is essential, and great progress has been made by applying these biomarkers in formulating individualized treatment plans for children with VVS. This review summarizes recent advances in predicting the effect of β-blockers in the management of VVS in children.

## 1. Introduction

Syncope is a common clinical symptom caused by transient cerebral blood supply reduction or interruption. It mainly manifests as transient loss of consciousness followed by decreased muscle tone and fall due to inability to maintain autonomous posture. The process is mostly temporary and self-limited (1). Neurally mediated syncope (NMS) is a very prevalent condition and accounts for a large proportion of pediatric syncopal cases, of which VVS is one of the most common forms (73%). In patients with VVS, sudden postural change from supine to upright, stuffy environments, and psychological stress lead to hypotension and/or bradycardia, which causes pre-syncope or syncope due to the inability to maintain posture. The physical and mental health of children with VVS is often affected (2, 3). In recent years, ample research has been conducted on individualized treatment of children with VVS (4). Currently, known therapeutic methods of VVS include pharmacological and non-pharmacological therapies. Beta-blockers, α1-adrenergic receptor agonist, sertraline, fludrocortisone, and oral rehydration salts are commonly used pharmacological treatments for VVS (1, 5, 6). This review summarizes the latest advances of biomarkers that can predict the therapeutic efficacy of β-blocker in children with VVS, and points out the advantages and disadvantages of these biomarkers. The aim of the present review is to describe the characteristics of VVS children who had a positive response to the treatment of β-blocker and to help the effective clinical use of β-blocker in the treatment of children with VVS.

## 2. Hypercatecholamine and hypersympathetic nerve function in children with VVS

The pathophysiology of VVS is very complex, involving a variety of potential mechanisms, such as hypercatecholamine and hypersympathetic nerve function, excessive vasorelexation or relatively low blood volume, etc (7–9). and the pathophysiology varies greatly among VVS individuals. In healthy children, when they stand for a long time or undergo sudden position changes from supine to upright, the decrease of the venous return results in a trend of declining blood pressure and heart rate; however by stimulating baroreceptors, they can be reflexively raised without causing obvious hypotension and symptoms. Previous studies have shown that the level of basal catecholamines in circulation of some VVS children is increased and they show hypersympathetic nerve function due to the imbalance of autonomic nervous function regulation. Therefore, in these children with VVS, due to the hypercatecholamine status and hypersympathetic nerve function, under the inducement factors such as sudden posture change, the Bezold-Jarisch reflex of the heart is initiated by the enhancement of compensatory myocardial contractility, which leads to the increase of vagus nerve activity and the heart rate and blood pressure continue to drop (10, 11). This causes insufficient blood supply to the brain, resulting in chest tightness, pallor, sweating, dizziness, blurred vision, and then fainting due to inability to maintain posture (12).

## 3. Treatment of beta-blockers in VVS of children

Patients with VVS are often treated with non-pharmacological and pharmacological therapies. Non-pharmacological therapy includes health education, fluid and salt supplementation, exercise of autonomic nervous function, and physical counter-pressure maneuvers. Pharmacological therapy includes β-blockers, α1-adrenergic receptor agonists, neurohormones, sertraline, fludrocortisone, and oral rehydration (13, 14). In clinical practice, the beta-blocker treatment for VVS was often prescribed empirically and non-selectively, and the efficacy of empirical treatment was often unsatisfactory. Previous studies have shown that the recurrence rate of syncope in children with VVS after 20 months of empirically non-selective metoprolol treatment was up to 34%, and the recurrence rate after 3 years was 43% (15, 16). Sheldon et al. found that there was no difference between metoprolol and placebo in preventing syncope in VVS patients (17).

## 4. Individualized beta-blocker therapy for children with VVS: biomarkers and therapeutic efficacy prediction

The empirical therapeutic effect of beta-blockers is often unsatisfactory because of the diversity of pathogenesis of VVS. According to recent studies, the pathogenesis of VVS includes increased sympathetic nerve activity and hypercatecholamine, a relatively insufficient central volume, peripheral vasodilation, neurohormonal disturbance, and the loss of baroreflex integrity. Therefore, the pathogenesis-based biological indicators should be determined to predict the therapeutic effect of drugs to implement individualized treatment to increase therapeutic efficacy (18). In some children with VVS, hypersympathetic nerve function and hypercatecholamine levels are the main mechanism, which can be antagonized by beta-blockers. Therefore, we should determine the stable biomarkers reflecting hypersympathetic nerve function and hypercatecholamine status as the main mechanism of VVS and direct β-blocker use as an individualized treatment to increase the therapeutic level (13). We summarizes the currently known biomarkers that can predict the efficacy of β-blockers on VVS in children (Table 1).

**Table 1:** Markers in predicting the therapeutic efficacy of beta-blockers in children with VVS.

References	Markers	Cut-off values	Sensitivity (%)	Specificity (%)
(19), Zhang et al.	ΔHR	30 bpm	81.0	80.0
(20), Song et al.	LVEF	70.5% (2 months after treatment)	80	100
		70.5% (6 months after treatment)	81.3	88.9
	LVFS	38.5% (2 months after treatment)	90.0	90.0
		37.5% (6 months after treatment)	93.8	66.7
(21), Tao et al.	BRS	10 ms/mmHg	71.0	83.0
	ΔBRS	4 ms/mmHg	81.3	88.9
(22), Kong et al.	24-h urinary NE	34.84 μg/24 h	70.0	100
(23, 24), Yuan et al.	L/T of Poincaré plot	2.7	88.2	82.8

ΔHR, the change of heart rate from supine to positive response occurrence during HUTT; LVEF, left ventricular ejection fraction; LVFS, left ventricular fractional shortening fraction; HUTT, head-up tilt test; BRS, baroreflex sensitivity; ΔBRS, the change of baroreflex sensitivity from supine to positive response occurrence during HUTT; NE, norepinephrine.

### 4.1. Changes in heart rate during the head-up tilt test (HUTT)

During the HUTT, some children with VVS develop reflective tachycardia after tilting from the supine position because of postural changes or long-term standing likely due to the sympathetic nerve activation (25, 26). Zhang et al. observed the hemodynamic changes of VVS children during HUTT and found that β-blockers were more effective for patients with significant heart rate increase before positive reaction during HUTT. A heart rate increase by 30 bpm before the positive response at HUTT as a cut-off value yielded a sensitivity of 81% and specificity of 80% to predict the responsiveness to β-blockers in VVS children (19).

### 4.2. Left ventricular ejection fraction (LVEF) and left ventricular fractional shortening fraction (LVFS)

Left ventricular ejection fraction (LVEF) and left ventricular fractional shortening fraction (LVFS) measured by echocardiography can reflect plasma catecholamine level and sympathetic nerve activity to some extent, although they are influenced by several other factors, such as cardiac function (27, 28). Based on the above correlations, Song et al. speculated that the VVS children with increased LVEF or LVFS might be in a status of high catecholamine level or sympathetic overexcitation, thereby exhibiting a better responsiveness to β-blocker (20). They followed up the VVS children treated with β-blocker for 6 months to explore the role of baseline LVEF and LVFS in the prediction of therapeutic efficacy of β-blocker in the children with VVS. As they expected, the results of follow-up at 6 months showed that the VVS children with baseline LVEF > 70.5% or LVFS > 37.5% responded better to β-blocker than those without, suggesting that the baseline LVEF and LVFS are meaningful and useful predictors of therapeutic response to β-blocker in children with VVS.

### 4.3. Baroreflex sensitivity

Baroreceptor reflex, as one of the important neuroregulatory reflexes to maintain blood pressure homeostasis, plays a key role in the regulation of hemodynamics when the body position changes. Abnormal changes in baroreceptors are one of the important pathogeneses of VVS. Measurements of baroreflex sensitivity (BRS) can evaluate the response to baroreceptors in the process of arterial pressure changes and reflect sympathetic nerve activity (6). The pre-treatment supine BRS of children with VVS to whom β-blockers are effective is much higher than that of children to whom β-blockers are ineffective. The change of BRS (ΔBRS) from supine position to positive response during the HUTT before treatment was more obvious in the effective patients than in the ineffective patients. Therefore, both BRS and ΔBRS can predict the response of VVS patients to β-blockers, and both indicators are non-invasive with good safety. However, the acquisition of these two indicators requires the use of specific instruments with relatively high cost, so they have not been widely used in clinics. Using pre-treatment BRS of 10 ms/mmHg as a cut-off value, the predicted sensitivity and specificity of β-blocker response in children with VVS were 82% and 83%, respectively. Pre-treatment ΔBRS of 4 ms/mmHg as a cut-off value yielded a sensitivity and specificity of 71% and 83%, respectively, to predict the efficacy of β-blockers in children with VVS (21).

### 4.4. Twenty four-hour urinary norepinephrine

Norepinephrine (NE) is released from adrenergic nerve endings mainly as a neurotransmitter and reflects the sympathetic nerve activity to some extent. NE is excreted through urine. Therefore, the measurement of NE at 24-h would reflect sympathetic nerve activity to some extent (9). The 24-h urinary NE concentration has the advantages of being relatively stable and inexpensive. Studies have shown that VVS patients with high 24-h urinary NE levels before treatment exhibit better β-blocker treatment effects. Pre-treatment 24-h urinary NE of 34.84 μg/24 h as a cut-off value yielded a sensitivity and specificity of 70% and 100%, respectively, to predict the responsiveness to β-blockers in children with VVS (22).

### 4.5. L/T of poincaré plot

The Lorenz-RR scatterplot, also called the Poincaré plot, is converted from 24-h dynamic electrocardiogram monitoring data and used to evaluate autonomic nervous function (29). The longitudinal axis (L) of the Poincaré plot represents the 24-h heart rate variation and reflects the sympathetic tension to some extent. The longer the longitudinal axis is, the stronger is the sympathetic nerve activity. The transverse axis (T) reflects the difference among adjacent RR segments, which represents the instantaneous heart rate change and mainly reflects vagal tone (24). According to previous studies, the L/T of the Poincaré plot is also called the “cardiac sympathetic index” because it reflects cardiac sympathetic function. The L/T value is stable, intuitive, and non-invasive. Although it is time-consuming in detection, it is easier to obtain than the 24-h urinary NE concentration. Studies have shown that when children with VVS were treated with β-blockers, the L/T in patients of the effective group before treatment was higher than that in the ineffective group. A pre-treatment L/T of 2.7 as a cut-off value yielded a sensitivity and specificity of 88.2% and 82.8%, respectively, to predict the efficacy of β-blockers in children with VVS (23).

### 4.6. Beta-1 receptor gene polymorphism

Adrenergic receptors (ARs) are targeted by epinephrine and norepinephrine in the sympathetic nervous system. They are expressed on almost all types of cells and are important parts of the sympathetic nervous system. Therefore, they play an important role in maintaining homoeostasis. The abnormal changes of structural and functional ARs are closely related to the pathogenesis of many diseases. There are nine subtypes of human ARs: α1A, α1B, α1D, α2A, α2B, α2C, β1, β2, and β3 (30). Stimulation of the cardiac β1 receptor can produce positive chronotropic, inotropic, and dromotropic effects through G protein coupled signaling pathways. Adenylate cyclase is a second messenger in the G-protein coupled signaling pathway. Mason et al. found that nine adrenergic genes have polymorphisms (31). β1 adrenocepter gene (ADBR1) encodes a functional protein with 477 amino acids, in which there are multiple single nucleotide polymorphism (SNPs). According to the nucleotide variant #1165, the amino acid position of 389 can encode arginine and glycine. The Arg389 variant is more effective in stimulating adenylate cyclase than Gly389 variant, and has a better response to catecholamine. Therefore, the signal transduction ability of Arg389 variant is more stronger than Gly389 variant (32). The imbalance of autonomic nervous system regulation is one of an important pathogenesis of patients with VVS, and ADBR1 polymorphism may lead to changes in the structure and function of β1 adrenoceptor, and then result in autonomic nervous system imbalance. Beta-blockers can antagonize the binding of catecholamines to β1 adrenoceptor and block signal transduction, thus playing a role in the treatment of VVS children. In recent years, in the study of the role of β1 adrenoceptor gene polymorphism in VVS, it was found that the patients of VVS with positive HUTT had a higher frequency of Gly389 variant than VVS patients with negative HUTT (33.33% vs. 14.58%) (33). Atici et al. found that patients of VVS with Arg389Arg variant had a higher frequency of syncope episodes before β-blocker treatment than those with Arg389Gly variant (7.9 ± 3.7 vs. 6.4 ± 3.0). After 18 months of beta-blocker treatment, the number of syncope episodes of VVS patients with Arg389Arg variant was significantly lower than that of Arg389Gly variant (3.0 ± 1.4 vs. 6.8 ± 3.2) (32). However, the detection of β1 receptor gene polymorphism is a complex and costly process, and it is mainly used for research, as its wide application in clinical practice is not viable.

## 5. Conclusion and perspective

In summary, the markers including hemodynamic data during HUTT, characteristics of electrocardiogram, echocardiographic parameters, genetic information, and laboratory biochemistry index were successively found to have the ability to predict the therapeutic response to β-blocker in children with VVS. The facts that the above baseline clinical indices were collected before the treatment made it possible to help the pediatricians early identify the VVS children responding to β-blocker and then select the effective therapy. However, most of markers discovered so far have their own limitations such as unstability, unworkability, and expensiveness. In the future, the ability of markers to predict therapeutic response to β-blocker still needs to be improved. Moreover, it is worthy of paying great attention to the external validation study. Finally, the more novel, convenient, inexpensive, and readily available markers are still expected.
